# Fluorescence-aided caries excavation in minimally invasive dentistry: a scoping review

**DOI:** 10.1007/s00784-026-07119-9

**Published:** 2026-09-10

**Authors:** Marco M. Herz, Johannes Österreicher, Christian Meller

**Affiliations:** Department for Conservative Dentistry, University of Tübingen, Osianderstr. 2-8, Tuebingen, 72076 Germany

**Keywords:** Fluorescence-guided caries excavation, Fluorescence-aided caries excavation (FACE), Dentin caries removal, Minimally invasive dentistry, Fluorescence imaging, Operative dentistry

## Abstract

**Objective:**

The aim of this scoping review was to systematically map the available evidence on fluorescence-aided caries excavation (FACE) and related fluorescence-based technologies used to guide dentin caries removal in operative dentistry.

**Data sources:**

A comprehensive literature search was conducted in PubMed/MEDLINE, Embase, CINAHL, LIVIVO, the Cochrane Library, Web of Science Core Collection, and BIOSIS Citation Index for studies published between January 1, 2000 and June 1, 2026.

**Study selection:**

Studies investigating fluorescence-based technologies used intraoperatively to guide dentin caries excavation were included. In vitro, ex vivo, and clinical studies were considered eligible. Studies focusing exclusively on fluorescence-based caries detection without an excavation component were excluded. Two reviewers independently screened titles, abstracts, and full texts according to predefined eligibility criteria.

**Results:**

A total of 16 studies were included, predominantly laboratory-based (12 in vitro, 1 ex vivo), with only three clinical studies. Across the included studies, fluorescence-guided excavation generally demonstrated enhanced selectivity, enabling preservation of less mineralized dentin while maintaining effective removal of bacterially infected tissue, although these advantages were not observed uniformly across all studies. Microbiological analyses showed reduced residual bacterial contamination following FACE compared with conventional excavation and dye-assisted methods. In addition, fluorescence signals were found to correlate with bacterial presence and diversity, supporting their biological relevance. Clinical studies indicated improved intraoperative detection of residual caries and provided biologically relevant information on bacterial contamination. However, the evidence base was heterogeneous and primarily focused on surrogate outcomes, with limited data on long-term clinical performance.

**Conclusion:**

Fluorescence-guided caries excavation represents a promising adjunctive approach that provides qualitative intraoperative visualization of fluorescence characteristics associated with bacterial contamination, rather than a validated quantitative threshold for tissue removal. While the available evidence suggests improved excavation selectivity and microbiological outcomes, robust clinical data remain limited. Further well-designed clinical studies are required to determine the impact of FACE on patient-centered outcomes and its role in routine operative dentistry.

**Clinical relevance:**

Fluorescence-guided technologies may assist clinicians in identifying bacterially infected dentin during cavity preparation and facilitate selective caries removal, potentially improving intraoperative decision-making.

**Supplementary Information:**

The online version contains supplementary material available at https://doi.org/10.1007/s00784-026-07119-9.

## Highlights

Fluorescence-guided excavation enables visualization of bacterially contaminated dentin based on endogenous fluorescence signals.

Laboratory evidence suggests that FACE-based approaches may improve excavation selectivity while preserving less mineralized dentin.

Fluorescence signals correlate with bacterial presence and support microbiologically guided caries removal.

Current evidence is predominantly laboratory-based, with limited clinical validation.

Further well-designed clinical studies are required to determine long-term clinical effectiveness.

**Supplementary Information:**

The online version contains supplementary material available at https://doi.org/10.1007/s00784-026-07119-9.

## Introduction

Contemporary operative dentistry increasingly follows the principles of minimally invasive treatment, aiming to remove bacterially infected dentin while preserving affected tissue in order to maintain tooth vitality and structural integrity [1–3]. Selective caries removal has therefore become a key concept in modern caries management, as it seeks to eliminate infected dentin while preserving dentin that retains the potential for remineralization and pulpal protection [3, 4].

Determining the appropriate endpoint of dentin caries excavation therefore remains one of the central challenges in restorative dentistry. Excessive removal may lead to unnecessary loss of tooth structure or pulp exposure, whereas insufficient excavation may result in residual infected dentin and potential restoration failure [2, 5].

Traditionally, and still widely in current clinical practice, clinicians rely on visual inspection and tactile feedback using hand instruments such as spoon excavators and dental explorers to guide caries removal. However, these approaches are inherently subjective and may not reliably differentiate between bacterially infected dentin and affected dentin that could potentially be preserved [6].

To improve the objectivity of intraoperative caries detection, several adjunctive technologies have been developed to assist clinicians during caries excavation [6]. Among these, fluorescence-based techniques have gained considerable attention because they uniquely allow visualization of bacterial metabolites within carious dentin, thereby supporting more selective caries removal [7].

The principle of fluorescence detection is based on the presence of endogenous fluorophores, particularly bacterial porphyrins, which emit red–orange fluorescence when excited with blue-violet light [7]. Porphyrin derivatives implicated in this fluorescence include protoporphyrin IX and related porphyrin compounds; however, the relative contribution of individual fluorophores may vary depending on the microbial composition and lesion environment. In addition, alterations of the dentin matrix and mineral content may modify the overall fluorescence intensity and spectral characteristics of carious dentin, although the red–orange fluorescence used for FACE is primarily associated with bacterial metabolites rather than serving as a direct measure of matrix degradation [7]. Although red–orange fluorescence has been associated with bacterial contamination, this signal should not be interpreted as a definitive indicator that the respective dentin requires removal, particularly in deep lesions where selective caries removal is recommended [2, 4].

In contrast, affected dentin typically appears as a darkened area with reduced or absent green fluorescence, whereas sound dentin exhibits intense green fluorescence. This optical contrast provides a visual adjunct for intraoperative assessment but does not define a strict histological boundary between infected and affected dentin or a quantitative excavation threshold. Based on these observations, fluorescence-aided caries excavation (FACE) was introduced in the early 2000s as a technique designed to assist clinicians in identifying fluorescence patterns associated with bacterial contamination during operative procedures [8].

**Fig. 1:**
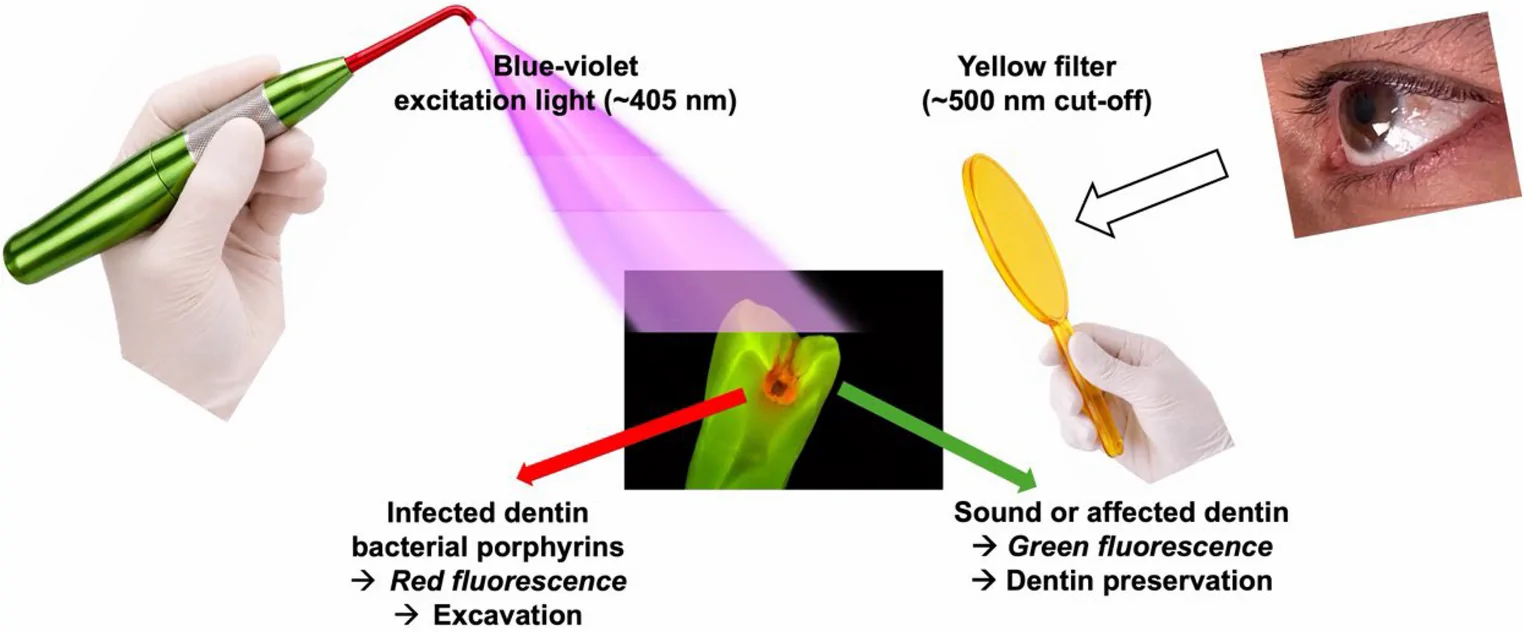
Principle of fluorescence-guided dentin caries excavation. Blue-violet light (~ 405 nm) excites endogenous fluorophores, including bacterial porphyrins, producing red–orange fluorescence associated with bacterial contamination. Fluorescence patterns provide adjunctive intraoperative information but should not be interpreted as defining a strict boundary between infected and affected dentin or as a quantitative endpoint for tissue removal

In this approach, blue-violet excitation light in the wavelength range of approximately 370–420 nm is directed onto the dentin surface, as illustrated in Fig. 1, while yellow or orange filters allow the visualization of the red–orange fluorescence emitted from bacterial metabolites within the carious tissue [7, 8].

Three fluorescence patterns have commonly been described [7, 8]:

1. areas exhibiting intense red/orange fluorescence associated with increased bacterial contamination;
2. darkened areas with reduced or absent green fluorescence; and.
3. areas exhibiting intense green fluorescence, typically associated with sound dentin.

**Fig. 2:**
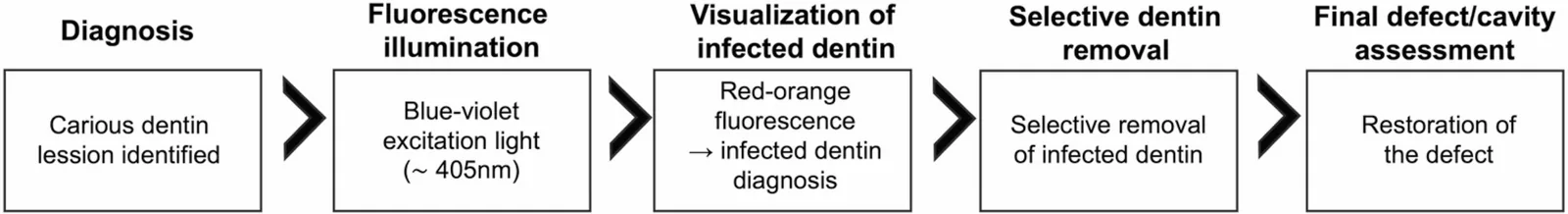
Clinical workflow of fluorescence-guided dentin caries excavation

Early experimental investigations revealed that fluorescence-guided excavation could improve the detection of infected dentin compared with conventional excavation methods based solely on visual and tactile criteria [8]. Subsequent studies evaluating FACE in both permanent and primary teeth reported that fluorescence-guided excavation may enhance the identification of residual caries and support a more conservative removal of dentin tissue [9, 10]. Figure 2 illustrates the clinical workflow of fluorescence-guided dentin caries excavation.

Further experimental studies have investigated the diagnostic and microbiological validity of fluorescence signals during caries excavation. Red–orange fluorescence has been shown to correlate with bacterial contamination and cariogenic activity, suggesting that fluorescence signals may serve as an indicator of infected dentin during operative procedures [11, 12]. These findings support the use of fluorescence as an adjunctive method for guiding caries removal within the framework of minimally invasive dentistry. Importantly, FACE should be distinguished from fluorescence-based technologies developed primarily for caries detection. FACE typically uses violet–blue excitation light at approximately 405 nm to visualize red–orange fluorescence associated with bacterial metabolites during excavation [7, 9]. In contrast, laser fluorescence devices such as DIAGNOdent use excitation at approximately 655 nm and provide numerical diagnostic readings rather than direct visual guidance of the excavation process [13]. Camera-based fluorescence systems may use different excitation and detection principles and are primarily intended for lesion detection or assessment, although some have also been investigated in an intraoperative context. More recent investigations have also explored the potential role of fluorescence-guided excavation in combination with other minimally invasive techniques, including chemo-mechanical caries removal methods, further highlighting the clinical relevance of fluorescence-based feedback during caries excavation procedures [12].

In addition to experimental investigations, several studies have evaluated fluorescence-guided technologies for intraoperative caries detection during selective caries removal. Evidence suggests that fluorescence-based systems may demonstrate higher sensitivity and specificity in detecting infected dentin compared with traditional caries detector dyes or purely visual-tactile methods [14]. These findings indicate that fluorescence-guided excavation may support clinicians in achieving a more reliable differentiation between infected and remineralizable dentin during operative treatment.

Despite these promising developments, the available body of evidence on fluorescence-guided dentin caries excavation remains heterogeneous. Most investigations have been conducted under laboratory conditions using extracted teeth, whereas relatively few clinical studies have evaluated fluorescence-guided excavation in routine dental practice. In addition, different fluorescence devices and methodological approaches have been investigated, making it difficult to obtain a comprehensive overview of the technologies used and their reported outcomes in caries excavation procedures.

Therefore, the aim of the present scoping review is to systematically map the existing evidence on fluorescence-based technologies used to guide dentin caries excavation in operative dentistry, with a particular focus on fluorescence-aided caries excavation (FACE) and related fluorescence-guided approaches. The review seeks to map the available evidence regarding diagnostic performance, microbiological outcomes, dentin preservation, and reported clinical applications of fluorescence-guided excavation technologies, while considering the predominantly preclinical nature of the current evidence base.

## Methods

### Protocol and reporting guidelines

This scoping review was conducted in accordance with the methodological framework proposed by Arksey and O’Malley and further refined by Levac et al. The review is reported following the Preferred Reporting Items for Systematic Reviews and Meta-Analyses extension for Scoping Reviews (PRISMA-ScR) guidelines [15]. The completed PRISMA-ScR checklist is provided in Supplementary Table S1. The review protocol was prospectively registered with the Open Science Framework (OSF) on March 15, 2026, prior to the commencement of study screening (OSF Preregistration; registration identifier: nkz6v; DOI: 10.17605/ OSF.IO/NKZ6V), and was publicly accessible from the date of registration. The registration was subsequently updated on April 16, 2026. Deviations from the registered protocol included extension of the final literature search from March 10 to June 1, 2026 to capture more recent evidence and expansion of language eligibility from English only to English and German. No other substantive deviations from the registered methodology occurred.

### Eligibility criteria

Studies were included if they met the following criteria:

- investigated fluorescence-aided caries excavation (FACE) or any other fluorescence-guided method for caries excavation/removal,
- involved human teeth (in vitro, ex vivo, or in vivo), and.
- reported outcomes related to caries removal, bacterial presence, dentin properties, diagnostic performance, or clinical parameters.

To meet the operative component of the eligibility criteria, fluorescence had to be applied during the caries excavation procedure to guide, assess, or determine tissue removal. Studies in which fluorescence was used exclusively for pre- or post-operative caries detection without influencing or assessing the excavation procedure were not considered eligible.

Studies were excluded if they:

- were published before January 1, 2000,
- did not involve fluorescence-guided excavation (e.g., fluorescence used solely for diagnostic purposes),
- did not report relevant outcomes related to caries excavation, or.
- were not original research articles (e.g., reviews, book chapters, case reports).

Studies published in English or German were considered eligible. Grey literature, conference abstracts and proceedings, dissertations, and theses were excluded; inclusion was restricted to original research articles. No restrictions were applied regarding study design or patient population.

### Information sources

A comprehensive literature search was conducted in the following electronic databases: PubMed/MEDLINE, Embase, CINAHL, LIVIVO, Cochrane Library, Web of Science Core Collection, and BIOSIS Citation Index. These databases were selected to ensure broad coverage of biomedical, dental, and life sciences literature relevant to fluorescence-based technologies for caries excavation.

All electronic databases were searched on June 1, 2026.

### Search strategy

The search strategy was designed to identify studies investigating fluorescence-based technologies specifically applied to dentin caries excavation. To maintain specificity, broader fluorescence-related terms used solely for caries detection without an excavation component were excluded.

The electronic database searches were conducted without a publication-date restriction. The predefined eligibility criterion restricting inclusion to studies published from January 1, 2000 onwards was subsequently applied during title and abstract screening. Search strategies were adapted for each database using controlled vocabulary (e.g., MeSH terms) and database-specific syntax. The full search strategies for all databases are provided in Supplementary Table S2. Additionally, reference lists of included studies were screened for relevant publications (backward citation searching). Systematic forward citation searching was not performed.

### Study selection

All records identified through database searches were imported into EndNote reference management software (Clarivate Analytics, Philadelphia, PA, USA) for automatic deduplication. Additional duplicates were identified and removed manually.

Following deduplication, records were exported to Microsoft Excel (Microsoft Corporation, Redmond, WA, USA) to facilitate screening. Study selection was conducted in two stages. In the first stage, titles and abstracts were independently screened by two reviewers according to the predefined eligibility criteria. In the second stage, full texts of potentially eligible studies were retrieved and independently assessed by the same reviewers.

Full-text articles were evaluated based on population, concept, and context. Reasons for exclusion at the full-text stage were documented and categorized. Disagreements between reviewers were resolved through discussion, and a third reviewer was consulted when necessary. Inter-rater agreement was not formally quantified because agreement statistics were not prespecified as part of the scoping review methodology; disagreements were instead resolved through consensus discussion, with consultation of a third reviewer when necessary. All reviewers had prior experience in the subject area.

The study selection process is presented in a PRISMA flow diagram.

### Data charting

A standardized data charting form was developed in Microsoft Excel to extract relevant data from included studies. The following variables were recorded: author and year of publication, country, study design, study setting (in vitro, ex vivo, or in vivo), fluorescence device type used (prototype or commercial device) with corresponding wavelength parameters, sample characteristics and size, outcome measures, and key findings. Data charting was performed by one reviewer and verified by a second reviewer to ensure accuracy and completeness.

### Critical appraisal of individual sources of evidence

Consistent with the objectives of scoping reviews, no formal assessment of methodological quality or risk of bias was undertaken, in line with established methodological guidance [16]. Consequently, the findings of this review should be interpreted with caution, particularly when drawing clinical implications.

### Data synthesis

Due to the heterogeneity in study designs, fluorescence technologies, and outcome measures, a quantitative synthesis was not performed. Instead, results were synthesized descriptively using a narrative approach.

Studies were grouped according to study design, type of fluorescence technology with its corresponding wavelength parameters, and reported outcomes related to dentin caries excavation.

### Presentation of results

The study selection process is presented using a PRISMA flow diagram. Characteristics of included studies are summarized in tabular form. Findings are reported thematically according to fluorescence technologies and clinical or experimental outcomes related to fluorescence-guided caries excavation.

## Results

### Study selection

**Fig. 3:**
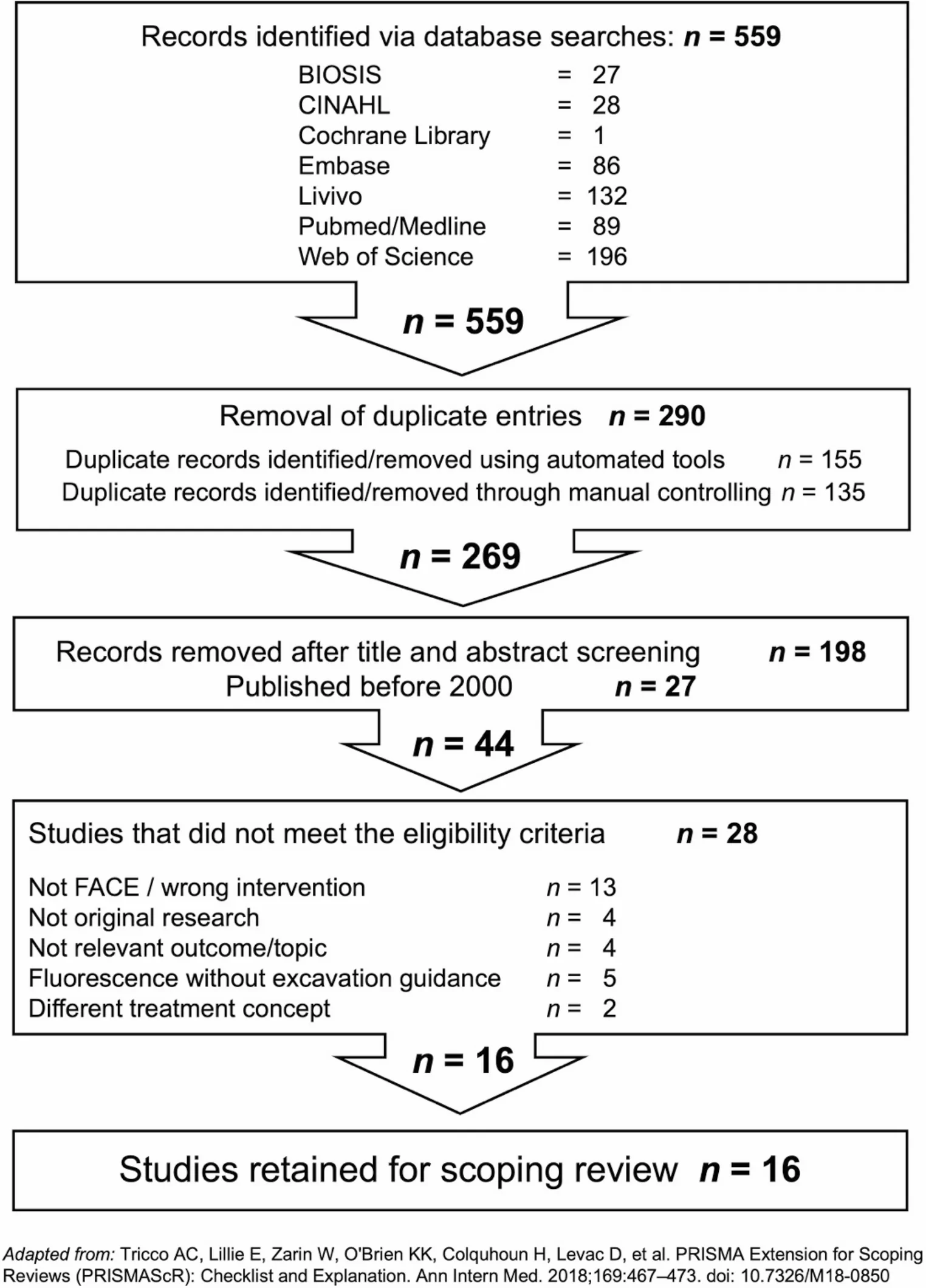
PRISMA-ScR Flow Chart

A total of 16 studies were included in this scoping review. The literature search initially identified 559 records. After removal of 155 duplicates using reference management software and an additional 135 duplicates identified through manual screening, 269 records remained for title and abstract screening. Of these, 27 records were excluded due to publication before the predefined cut-off date of January 1, 2000, and 198 records were excluded based on title and abstract screening as they did not meet the inclusion criteria. This resulted in 44 full-text articles assessed for eligibility. Following full-text review, 28 articles were excluded, leaving 16 studies for inclusion in the final analysis. The study selection process is illustrated in Fig. 3.

### Study characteristics

The majority of the included studies were conducted under laboratory conditions, with twelve studies employing in vitro designs. In addition, three studies were performed in clinical (in vivo) settings, involving both adult and pediatric populations, while one study used an ex vivo design combining experimental procedures with microbiological analysis. Sample sizes varied considerably, ranging from approximately 20 extracted teeth in laboratory studies to more than 400 cavities in clinical cohorts (Table 1).

Geographically, the included studies were predominantly conducted in Europe, particularly in Germany, Switzerland, and Turkey, with additional contributions from Asia, mainly China, and only limited representation from North America. This distribution indicates a strong European focus in research on fluorescence-guided caries excavation.

The included studies investigated fluorescence-based approaches for caries detection during excavation. Where reported, violet-blue excitation wavelengths were typically within the range of approximately 370–420 nm, most commonly around 405 nm.

The most frequently used devices included SIROInspect (Sirona Dental Systems GmbH, Bensheim, Germany), FaceLight (W&H Dentalwerk Bürmoos GmbH, Bürmoos, Austria), and the camera-based VistaProof system (Dürr Dental AG, Bietigheim-Bissingen, Germany). These systems differ in their mode of use, with SIROInspect and FaceLight, which are technically equivalent systems, designed for direct intraoral visualization, while VistaProof relies on camera-based imaging and software-assisted analysis.

A comprehensive overview of all devices applicable for FACE and similar systems is provided in Supplementary Table S3A and S3B.

Conventional excavation served as the primary comparator in most studies, while additional comparators included caries detector dyes, chemomechanical excavation methods, and fluorescence-based diagnostic devices. Overall, the current body of evidence is characterized by a predominance of laboratory-based studies, methodological heterogeneity, and a focus on surrogate outcomes, whereas long-term clinical data and independent validation remain limited. A structured evidence map of the included studies, including study design, sample and tooth type, fluorescence technology and excitation wavelength, comparator, primary outcome, principal findings, and relevant study-specific limitations, is provided in Table 1. All technologies included in the evidence synthesis were investigated in an operative context in which fluorescence was used to guide or assess carious tissue removal; devices evaluated exclusively for diagnostic caries detection were excluded according to the predefined eligibility criteria.

**Table 1:** Evidence map of included studies on fluorescence-aided caries excavation (FACE)

Study	Design / sample / tooth type	FACE technology / excitation	Comparator(s)	Primary outcome	Principal findings	Relevant limitations
Lennon et al. [13]	In vitro; *n* = 40 teeth with D2 caries; tooth type NR	Visible-fluorescence prototype; violet-blue excitation + 530-nm high-pass filter; exact excitation wavelength NR	Visual, CDD, DIAGNOdent	Diagnostic performance	Visible fluorescence showed the highest sensitivity and specificity for residual caries detection and provided proof-of-principle for FACE.	Laboratory study; diagnostic surrogate outcome; exact excitation wavelength NR
Lennon [8]	In vitro; *n* = 40 extracted teeth; tooth type NR	Integrated FACE prototype; 370–420 nm + 530-nm high-pass filter	CE	Residual caries / microbiology	FACE resulted in fewer residual caries lesions than CE (3 vs. 9; *p* = 0.037).	Laboratory study; microbiological surrogate outcome
Lennon et al. [17]	In vitro; *n* = 100; tooth type NR	FACE prototype; violet illumination; exact wavelength NR	CE, CD, CS	Excavation efficacy / time / bacteria	FACE was faster than chemomechanical and dye-assisted excavation and showed fewer residual bacteria.	Laboratory study; surrogate outcomes; exact wavelength NR
Lennon et al. [18]	In vitro; *n* = 60; tooth type NR	FACE prototype; violet light + high-pass filter; exact wavelength NR	CE, dye	Residual bacteria / cavity size	FACE reduced bacterial load without significantly increasing cavity size.	Laboratory study; surrogate outcomes; exact wavelength NR
Lennon et al. [10]	In vitro; *n* = 66 extracted primary teeth	FACE prototype; 370–420 nm + 530-nm filter	CE, dye	Histological assessment	FACE removed infected dentin in primary teeth more effectively than CE.	Laboratory study; histological surrogate outcome
Zhang et al. [19]	In vitro; carious molars; dentition NR	FACE; device and excitation wavelength NR	CE, CME, LIF	Micro-CT / tissue preservation	FACE showed a favorable balance between excavation efficacy and minimally invasive potential.	Device/optical parameters NR; micro-CT surrogate outcome
Ganter et al., [20]	In vitro; *n* = 100 C3 lesions; tooth type NR	VistaProof; camera-assisted fluorescence; exact excitation wavelength NR	CE, dye, visual-tactile	PCR / dentin preservation	Computer-assisted fluorescence resulted in low bacterial contamination while preserving dentin.	Laboratory study; surrogate outcomes; optical parameters incompletely reported
Lai et al. [21] – microhardness	In vitro; *n* = 20 extracted teeth; tooth type NR	FACE; device and excitation wavelength NR	CE	Microhardness	FACE preserved softer, less mineralized dentin than CE.	Device/optical parameters NR; laboratory surrogate outcome
Lai et al. [22] – micro-CT	In vitro; *n* = 20 extracted teeth; tooth type NR	FACE; device and excitation wavelength NR	CE	Mineral density / micro-CT	FACE left dentin with lower mineral concentration than CE.	Device/optical parameters NR; micro-CT surrogate outcome
Peskersoy et al. [23]	Clinical; *n* = 415 Class II cavities; molars/premolars	FaceLight (W&H); 405 nm	CDD	Residual caries detection	FACE identified suspicious residual areas more frequently than dye-based assessment.	Intraoperative detection endpoint; no long-term restorative outcome
Koç Vural et al. [9]	Clinical; *n* = 503 permanent teeth	FaceLight (W&H); 405 nm	Visual-tactile	Residual caries detection	FACE detected residual caries more frequently than visual-tactile assessment.	Detection endpoint; no long-term restorative outcome
Koç-Vural et al. [24]	In vitro; *n* = 48 specimens; human carious dentin	FaceLight (W&H); 405 nm	CE	Bond strength / microhardness	Bond strength after FACE was comparable to CE; FACE-treated dentin showed lower microhardness.	Laboratory study; mechanical surrogate outcomes
Trippe et al. [11]	Ex vivo; *n* = 21 teeth with deep dentin caries	SIROInspect (Sirona); 405 nm	Tactile assessment	Microbiome correlation	Red fluorescence correlated with bacterial diversity and load.	Ex vivo design; microbiological surrogate outcome
Aksoy et al. [25]	In vitro; *n* = 40 deciduous molars	FACE system; violet-blue excitation; device and exact wavelength NR	Different remaining dentin thicknesses	Pulp temperature	Temperature increased with exposure and decreasing remaining dentin thickness.	Device, irradiance and exact optical parameters NR; thermal surrogate outcome
Wang et al. [26]	In vitro; *n* = 30; molars/premolars	FACE; device and excitation wavelength NR	CE, CME	Micro-CT / excavation efficacy	FACE showed effective excavation with moderate invasiveness.	Device/optical parameters NR; micro-CT surrogate outcome
Lazarova et al. [12]	Clinical; *n* = 42 children; primary molars	ProFace (W&H); 405 nm, during Brix 3000 excavation	CME	Fluorescence intensity / bacterial load	Fluorescence intensity correlated strongly with cariogenic bacterial load.	Small clinical cohort; microbiological rather than long-term restorative outcome

FACE generally uses violet–blue excitation light, typically around 405 nm, to visualize fluorescence associated with bacterial metabolites during caries excavation. Exact excitation wavelengths and/or device specifications were not reported in several included studies and are indicated as NR

*Abbreviations and notes: FACE *Fluorescence-aided caries excavation, *CE *Conventional excavation, *CME *Chemomechanical excavation, *CDD *Caries detector dye, *CD *Caries detector, *CS *Carisolv, *LIF *Laser-induced fluorescence, *lab *Laboratory setting, *in vivo *Clinical setting, *ex vivo *Experimental setting using extracted teeth, *CRE *Caries removal efficacy, *MIP *Minimally invasive potential, *PCR *Polymerase chain reaction, *NR *Not reported

### Technical characteristics of FACE systems

The included studies investigated fluorescence-guided approaches generally based on violet-blue excitation and visualization of fluorescence associated with bacterial metabolites. Where reported, excitation wavelengths were typically around 405 nm; however, several studies did not provide sufficient device-specific or optical parameters. In systems for which optical parameters were reported, violet-blue excitation light, typically around 405 nm, was used to induce fluorescence associated with bacterial metabolites, particularly porphyrins. As a result, infected dentin emits red or orange fluorescence, whereas sound or less affected dentin appears green.

Despite this common mechanism, different devices were employed across studies. The most frequently used systems included SIROInspect (Sirona) and FaceLight (W&H), which are both technically identical devices marketed under different trade names and designed for intraoral application, as well as camera-based systems such as VistaProof (Dürr Dental), which allow for software-assisted visualization and analysis (Supplementary Table S3).

While these devices differ in terms of hardware and image processing, their diagnostic and operative principles remain comparable. Overall, findings across studies were generally consistent despite variations in device type.

Reporting of the fluorescence technology was incomplete in several studies. Specifically, Zhang et al. (2013), Lai et al. (2013), Lai et al. (2014), Aksoy et al. (2021), and Wang et al. (2024) did not specify the exact FACE device or light-source model used. This lack of standardized reporting of optical parameters, including the excitation source and device configuration, limits methodological reproducibility and complicates direct comparison of fluorescence-guided excavation outcomes across studies.

### Caries removal and tissue preservation

Across multiple in vitro studies, fluorescence-aided caries excavation (FACE) generally demonstrated a more conservative excavation pattern compared with conventional methods. Analyses using micro-computed tomography, mineral density assessment, and microhardness testing revealed that dentin remaining after FACE exhibited lower mineralization and reduced hardness values, indicating preservation of partially demineralized but structurally maintainable dentin. These laboratory findings suggest that FACE may support more selective dentin removal while preserving less mineralized dentin.

Despite this more conservative approach, FACE showed comparable or improved efficacy in removing infected dentin. Micro-CT–based studies demonstrated that FACE achieves a favorable balance between caries removal efficacy and minimally invasive potential. In comparative investigations, FACE performed at least as effectively as conventional excavation and showed superior performance compared to caries detector dyes and chemomechanical methods.

### Microbiological and diagnostic outcomes

Growing evidence supports the ability of fluorescence-based methods to detect and guide removal of bacterially infected dentin. An early in vitro study by Lennon et al. (2002) demonstrated that visible fluorescence achieved the highest sensitivity (94%), specificity, and overall diagnostic accuracy compared to visual-tactile examination, caries detector dye, and laser fluorescence devices [13]. This study provided foundational evidence for fluorescence-based detection of residual caries.

Building on this, Lennon (2003) demonstrated that fluorescence-aided caries excavation resulted in significantly fewer cases of residual caries compared to conventional excavation (3/20 vs. 9/20; *p* = 0.037), as assessed by confocal laser scanning microscopy [8]. Subsequent studies consistently confirmed reduced bacterial counts following FACE compared to conventional and dye-assisted excavation.

Subsequent microbiological studies showed strong correlations between fluorescence signals and bacterial presence, including cariogenic microorganisms such as Streptococcus mutans. The ex vivo study by Trippe et al. demonstrated that red-fluorescing dentin contained the highest bacterial diversity and load, supporting the biological validity of fluorescence-based methods [11].

Clinical studies further indicated that fluorescence-based approaches may enhance intraoperative detection of residual caries and provide biologically relevant information on bacterial contamination.

### Clinical performance and safety

Several studies evaluated procedural efficiency and reported that FACE was faster than chemomechanical excavation methods and dye-assisted techniques, while being comparable to or slightly faster than conventional excavation. The use of real-time fluorescence feedback may contribute to improved efficiency by reducing the need for repeated inspection.

With regard to mechanical outcomes, FACE-treated dentin exhibited lower microhardness values, consistent with preservation of affected dentin. However, bond strength measurements indicated that adhesive performance on FACE-treated dentin was comparable to that of conventionally excavated dentin, suggesting that FACE treatment did not adversely affect bond strength under the investigated laboratory conditions.

Safety-related outcomes were addressed in one study investigating intrapulpal temperature changes. Although the reported temperature increases remained below the historically cited threshold associated with pulpal injury, the lack of device-specific optical parameters precludes definitive conclusions regarding thermal safety.

## Discussion

The results of this scoping review provide a comprehensive overview of fluorescence-aided caries excavation (FACE) and highlight its potential role as an adjunctive tool in minimally invasive dentistry. Across the included studies, a consistent pattern emerges: fluorescence-guided excavation supports the selective removal of infected dentin while promoting preservation of tooth structure. It should be noted, however, that the current evidence base is largely derived from laboratory investigations. Despite the commercial availability of several fluorescence-based systems, clinical evidence evaluating FACE remains limited. The reasons for the scarcity of clinical studies cannot be determined from the available evidence. Consequently, the clinical effectiveness of FACE and its impact on long-term patient-centered outcomes remain insufficiently established.

### Principal findings

Across the included evidence, predominantly derived from laboratory studies, FACE showed potential to combine caries removal with enhanced tissue preservation. Several laboratory studies reported that dentin remaining after FACE exhibited lower microhardness and mineral density compared with conventional excavation, indicating preservation of affected rather than infected dentin [19, 21, 22]. At the same time, microbiological analyses indicated that this more conservative approach maintained effective caries removal, with several studies reporting reduced residual bacterial contamination following FACE compared to conventional excavation or dye-assisted methods [8, 17, 18, 20].

These findings are particularly relevant to current concepts in minimally invasive dentistry. Selective caries removal strategies aim to preserve affected – but not infected – dentin, while avoiding unnecessary dentin excavation and reducing the risk of pulpal exposure, which has been associated with improved clinical outcomes in deep lesions [2–5]. The results of the present review suggest that FACE may facilitate the clinical implementation of these principles by providing a biologically guided excavation endpoint based on fluorescence patterns associated with bacterial activity .

A key observation across multiple in vitro investigations is the increased selectivity of FACE compared to conventional excavation. Studies using microhardness testing and mineral density analysis consistently showed that FACE-treated dentin remained softer and less mineralized, reflecting preservation of affected dentin layers [21, 22]. Complementary micro-CT analyses further suggested that FACE may provide a favorable balance between caries removal efficacy and minimally invasive potential under laboratory conditions when compared with chemomechanical and conventional excavation techniques [19, 26].

In contrast, traditional excavation methods based on visual and tactile assessment are inherently subjective and may lead to overexcavation. Previous studies have highlighted the limitations of hardness- and color-based criteria, particularly in deep lesions where preservation of affected dentin is desirable [2, 3, 27]. Within this context, FACE offers a more objective approach by incorporating fluorescence signals linked to bacterial activity, potentially reducing operator-dependent variability.

The biological relevance of fluorescence signals represents one of the central strengths of the FACE concept. Early work by Lennon et al. (2002) demonstrated that visible fluorescence achieved superior diagnostic performance compared with the then conventional – yet still widely used – visual-tactile assessment, caries detector dyes [13]. Building on this, Lennon (2003) showed that fluorescence-guided excavation resulted in significantly fewer samples with residual caries compared to conventional excavation, as confirmed by confocal laser scanning microscopy [8]. Subsequent in vitro studies consistently reported lower residual bacterial load following FACE [17, 18, 20].

More recent investigations have provided further support for the microbiological validity of fluorescence signals. Trippe et al. (2020) demonstrated that orange-red-fluorescing dentin was associated with higher bacterial diversity and load, whereas non-fluorescing dentin corresponded to harder, less infected tissue [11]. Similarly, Lazarova et al. (2025) reported a strong correlation between orange-red fluorescence intensity and the presence of cariogenic bacteria during chemo-mechanical caries removal [12]. Together, these findings indicate that fluorescence-based guidance reflects biologically relevant tissue conditions rather than purely optical characteristics. Importantly, however, the association between red–orange fluorescence and bacterial contamination does not imply that all fluorescing dentin should be removed. The clinical interpretation of fluorescence should consider the location within the cavity and established principles of selective caries removal [2, 27]. At the peripheral cavity margins, excavation aims to provide sound hard tissue suitable for a reliable peripheral seal, whereas a more conservative approach is indicated at the deep pulpal wall to minimize the risk of pulp exposure and preserve pulpal vitality [2, 27]. Accordingly, residual red–orange fluorescence at the pulpal aspect of a deep lesion should not automatically prompt further excavation. Conversely, the current evidence does not support a specific fluorescence intensity or color threshold, such as complete elimination of red–orange fluorescence at peripheral margins or acceptance of a defined degree of orange fluorescence pulpal to the lesion. The optimal site-specific fluorescence-guided excavation endpoints therefore remain to be clinically validated.

Interestingly, discrepancies between fluorescence signals and tactile assessment were also observed. Trippe et al. (2020) found that some clinically hard dentin still exhibited orange-red fluorescence, suggesting that conventional criteria may underestimate residual bacterial contamination [11]. This finding supports the potential added value of fluorescence guidance in improving excavation accuracy.

Although FACE is primarily intended as an operative tool, several studies also demonstrated its diagnostic capabilities. Clinical investigations reported improved intraoperative detection of residual caries compared with conventional visual assessment [9]. These findings suggest that fluorescence-based approaches may enhance intraoperative decision-making, particularly in cases where the boundaries of dentin lesions are difficult to assess.

At the same time, it is important to clearly distinguish FACE from fluorescence-based diagnostic devices. During the study selection process, a substantial number of studies had to be excluded because fluorescence was used solely for detection rather than for guiding excavation. This highlights that the broader literature on fluorescence in dentistry is not directly transferable to FACE. Devices such as DIAGNOdent are likewise used for fluorescence-based caries detection, but they rely on a different excitation wavelength (~ 655 nm) and optical principle and were shown by Lennon et al. (2002) to be less suitable than FACE (~ 400 nm) for guiding excavation [13].

From a clinical perspective, the practical applicability of FACE is an important consideration. Evidence from comparative studies suggests that FACE is at least as efficient as conventional excavation and may be faster than chemomechanical or dye-assisted techniques [17, 26]. The use of real-time fluorescence feedback may reduce the need for repeated inspection and thereby streamline the excavation process.

Concerns regarding the mechanical properties of remaining dentin appear to be unfounded based on the available evidence. Although FACE-treated dentin showed lower microhardness values, adhesive bond strength was comparable to conventionally excavated dentin [24], indicating that preservation of affected dentin does not negatively affect immediate restorative performance [24].

Aksoy et al. (2021) reported intrapulpal temperature changes during fluorescence-aided excavation; however, the specific fluorescence device and relevant optical parameters were not sufficiently reported [25]. Consequently, these findings do not allow definition of device-specific thermal safety limits. Although a pulpal temperature increase of approximately 5.5 °C has historically been used as an experimental reference threshold for potential thermal injury [28], the available FACE literature does not provide sufficiently standardized data on irradiance and continuous exposure duration to derive a universally applicable safe exposure limit. As temperature changes may depend on factors such as light intensity, exposure duration, working distance, and illumination mode, the thermal safety of FACE cannot be considered fully established on the basis of the available evidence. Future studies should therefore report these parameters systematically to permit reproducible assessment of thermal safety.

### Limitations of the evidence base

Several limitations must be considered when interpreting the findings of this review. Most of the current evidence supporting FACE is derived from laboratory studies using surrogate outcome measures, including microbiological, microhardness, and micro-CT assessments, while clinical validation remains limited. Consequently, conclusions regarding long-term clinical outcomes cannot yet be reliably drawn. Second, considerable heterogeneity was observed across studies in terms of devices, comparators, and outcome measures, which precluded quantitative synthesis. Moreover, while microbiological and structural outcomes provide valuable insights, they cannot fully substitute for clinical endpoints such as restoration longevity or caries recurrence.

These limitations reflect broader challenges in the field of minimally invasive caries management. Although selective caries removal is widely recommended, variability in clinical implementation and uncertainty regarding optimal excavation endpoints persist [2, 29, 30].

Another limitation relates to the excavation protocol. Although selective caries removal is increasingly recommended to reduce the risk of pulp exposure, its biological predictability remains debated from an endodontic perspective [4, 27].

Residual infected dentin has been discussed as a potential source of persistent histological pulpal inflammation despite apparent clinical success. Therefore, the present findings should be interpreted with caution, as clinical asymptomaticity does not necessarily indicate complete histological resolution of pulpal inflammation or a favourable long-term pulpal prognosis.

Finally, the available evidence is derived from a relatively limited number of publications, many of which originate from a small number of closely interconnected research groups. This concentration of evidence may limit the independence and external validity of the available findings, as studies originating from closely related research groups may share methodological approaches, operator expertise, device configurations, and analytical procedures. Such clustering may also increase susceptibility to correlated reporting patterns or selective reporting; however, the present scoping review was not designed to assess publication or reporting bias or to determine whether effect estimates were systematically inflated. Independent replication by research groups not involved in the development or early evaluation of FACE is therefore warranted.

While this likely reflects the emerging and still niche character of fluorescence-guided excavation research, independent external validation across different institutions remains limited. Consequently, the generalizability and robustness of the current evidence should be interpreted with appropriate caution, and further studies from independent research groups are warranted.

Taken together, these limitations indicate that the exact clinical relevance of FACE cannot yet be considered fully established. In particular, robust evidence defining the precise fluorescence-guided excavation endpoint required to ensure long-term pulpal health is still lacking. Furthermore, it remains unclear whether orange-red fluorescence must be eliminated completely in all clinical situations. Therefore, further well-designed clinical studies are needed to validate the applicability and long-term clinical relevance of FACE-guided excavation thresholds across different carious scenarios.

### Preclinical surrogate outcomes versus clinical effectiveness

The distinction between preclinical surrogate outcomes and clinical effectiveness is particularly important when interpreting the current evidence on FACE. Most available studies assessed surrogate measures such as residual bacterial contamination, microhardness, mineral density, micro-CT-based tissue removal, or diagnostic accuracy [8, 10, 11, 13, 18–22, 24, 26]. While these outcomes provide important information on excavation selectivity and the biological relevance of fluorescence signals, favorable laboratory findings do not necessarily translate into superior long-term clinical outcomes. In particular, evidence remains insufficient to determine whether fluorescence-guided excavation improves clinically relevant endpoints such as pulpal vitality, restoration longevity, or caries recurrence compared with established selective caries removal strategies. Therefore, the promising preclinical findings should be interpreted as a rationale for further clinical investigation rather than as evidence of superior clinical effectiveness.

### Implications for research and practice

FACE may represent a useful adjunct to support a more biologically guided approach to caries excavation. By providing additional visual information beyond traditional tactile criteria, it may support selective caries removal while helping to preserve dentin that can potentially be maintained safely. This concept aligns with current principles of minimally invasive caries management aimed at preserving pulpal vitality and avoiding unnecessary tissue loss [2].

Taken together, the available evidence suggests that FACE may improve excavation accuracy by enabling visualization of bacterially contaminated dentin and reducing reliance on subjective tactile criteria [8, 11, 31].

However, fluorescence interpretation itself remains dependent on visual operator assessment. Future research should therefore aim to develop and validate objective fluorescence assessment criteria. Quantitative approaches based on fluorescence intensity, spectral characteristics, or chromaticity scoring may facilitate more standardized interpretation across operators and devices. Such scoring systems should be validated against biological reference standards and clinically relevant outcomes before they can be used to define excavation thresholds.

Future research should focus on well-designed laboratory and in vivo clinical trials comparing FACE with established selective caries removal strategies. In particular, studies should evaluate the biological necessity of removing all orange-red fluorescing dentin to determine its impact on patient-centered outcomes such as pulpal vitality, restoration longevity, and caries recurrence, as well as practical aspects including treatment time and cost-effectiveness. Such evidence is essential to determine whether the advantages observed in laboratory settings translate into meaningful clinical benefits.

## Conclusion

This scoping review indicates that fluorescence-guided caries excavation, particularly fluorescence-aided caries excavation (FACE), represents a promising adjunctive approach for supporting selective carious tissue removal within the principles of minimally invasive dentistry. The available evidence suggests that fluorescence guidance may improve the identification and removal of bacterially infected dentin while facilitating preservation of affected dentin that may be maintained safely.

The biological rationale for FACE is supported by consistent associations between fluorescence signals and bacterial contamination, and the current evidence indicates favorable microbiological and tissue-preserving outcomes. However, the available literature is dominated by laboratory investigations, and robust clinical evidence demonstrating improved long-term patient-centered outcomes remains limited.

Further well-designed prospective clinical studies are required to determine whether fluorescence-guided excavation translates into improved pulpal health, restoration longevity, and reduced caries recurrence, and to establish evidence-based clinical criteria for its routine application. Until such evidence becomes available, FACE should be regarded as a promising adjunctive technology rather than a replacement for established principles of selective caries removal.

Future clinical research should prioritize the development and validation of standardized, site-specific fluorescence-guided excavation thresholds before FACE can be reliably integrated into routine clinical decision-making.

## Supplementary Information

Supplementary Material 1.

## Data Availability

No datasets were generated or analysed during the current study.
