# Supplementary material for: Fluorescence-aided caries excavation in minimally invasive dentistry: a scoping review
Source: Clin Oral Investig. 2026 Sep 10;30(10):440. doi: 10.1007/s00784-026-07119-9 (PMC13562286; doi:10.1007/s00784-026-07119-9)
Supplement: Supplementary file 1 [file 784_2026_7119_MOESM1_ESM.docx]

**Supplementary Table S1:** Preferred Reporting Items for Systematic reviews and Meta-Analyses extension for Scoping Reviews (PRISMA-ScR) Checklist

| **Section** | **Item** | **Prisma-ScR Checklist Item** | **Reported On Page #** |
| --- | --- | --- | --- |
| **Title** | 1 | Identify the report as a scoping review. | Title page (p. 1) |
| **Abstract** | 2 | Structured summary including background, objectives, eligibility criteria, sources of evidence, charting methods, results, and conclusions. | Abstract (pp. 1–2) |
| **Introduction** | 3 | Describe rationale for the review and justify scoping approach. | Introduction (pp. 2–4) |
|  | 4 | Explicit statement of objectives with PCC elements. | End of Introduction (p. 4) |
| **Methods** | 5 | Protocol and registration. | Section 2.1 (pp. 4–5) |
|  | 6 | Eligibility criteria with rationale. | Section 2.2 (p. 5) |
|  | 7 | Information sources and date of last search. | Section 2.3 (p. 5) |
|  | 8 | Full electronic search strategy for at least one database. | Section 2.4 (pp. 5–6); Supplementary Table S2 |
|  | 9 | Selection process (screening and eligibility). | Section 2.5 (p. 6); Figure 3 (p. 8) |
|  | 10 | Data charting process. | Section 2.6 (p. 6) |
|  | 11 | Data items extracted. | Section 2.6 (p. 6) |
|  | 12 | Critical appraisal rationale and method (if applicable). | Section 2.7 (p. 6) |
|  | 13 | Methods of synthesis. | Section 2.8 (p. 6) |
| **Results** | 14 | Numbers screened, assessed, included; reasons for exclusion; flow diagram. | Section 3.1 (p. 8); Figure 3 (p. 8) |
|  | 15 | Characteristics of included sources. | Section 3.2 (pp. 9–11); Table 2 (pp. 9–11) |
|  | 16 | Critical appraisal results (if done). | Not applicable |
|  | 17 | Results of individual sources of evidence. | Sections 3.3–3.6 (pp. 11–12); Table 2 (pp. 9–11) |
|  | 18 | Summary of charting results in relation to objectives. | Sections 3.2–3.6 (pp. 9–12) |
| **Discussion** | 19 | Summary of evidence linked to objectives and relevance. | Sections 4.1–4.4 (pp. 13–17) |
|  | 20 | Limitations of the review process. | Section 4.2 (pp. 15–16) |
|  | 21 | Conclusions and implications. | Section 5 (p. 17) |
| **Funding** | 22 | Funding sources and role of funders. | Funding statement — page to be confirmed |

**Supplementary Table S2:** Complete electronic search strategies for all databases

| **Database** | **Search strategy** |
| --- | --- |
| **PubMed/ MEDLINE** | ("dental caries"[MeSH Terms] OR caries[Title/Abstract] OR "dentin caries"[Title/Abstract] OR "carious dentin"[Title/Abstract]) AND (fluorescence[Title/Abstract] OR "fluorescence-guided"[Title/Abstract] OR "fluorescence-aided"[Title/Abstract] OR "fluorescence-aided caries excavation"[Title/Abstract] OR FACE[Title/Abstract] OR FIT[Title/Abstract] OR SIROInspect[Title/Abstract]) AND (excavat*[Title/Abstract] OR "caries removal"[Title/Abstract] OR "dentin removal"[Title/Abstract] OR "selective caries removal"[Title/Abstract]) |
| **Embase** | ('dental caries'/exp OR caries:ti,ab OR 'dentin caries':ti,ab OR 'carious dentin':ti,ab) AND (fluorescence:ti,ab OR 'fluorescence guided':ti,ab OR 'fluorescence aided':ti,ab OR 'fluorescence aided caries excavation':ti,ab OR face:ti,ab OR "fluorescence induced technique":ti,ab OR siroinspect:ti,ab) AND (excavat*:ti,ab OR 'caries removal':ti,ab OR 'dentin removal':ti,ab OR 'selective caries removal':ti,ab) |
| **CINAHL** | (MH "Dental Caries" OR TI caries OR AB caries OR TI "dentin caries" OR AB "dentin caries" OR TI "carious dentin" OR AB "carious dentin") AND (TI fluorescence OR AB fluorescence OR TI "fluorescence guided" OR AB "fluorescence guided" OR TI "fluorescence aided" OR AB "fluorescence aided" OR TI "fluorescence aided caries excavation" OR AB "fluorescence aided caries excavation" OR TI FACE OR AB FACE OR TI FIT OR AB FIT OR TI SIROInspect OR AB SIROInspect) AND (TI excavat* OR AB excavat* OR TI "caries removal" OR AB "caries removal" OR TI "dentin removal" OR AB "dentin removal" OR TI "selective caries removal" OR AB "selective caries removal") |
| **Web of Science Core Collection** | TS=(caries OR "dentin caries" OR "carious dentin") AND TS=(fluorescence OR "fluorescence guided" OR "fluorescence aided" OR "fluorescence aided caries excavation" OR FACE OR FIT OR SIROInspect) AND TS=(excavat* OR "caries removal" OR "dentin removal" OR "selective caries removal") |
| **Cochrane Library** | (caries OR "dentin caries" OR "carious dentin") AND (fluorescence OR "fluorescence guided" OR "fluorescence aided" OR "fluorescence aided caries excavation" OR FACE OR FIT OR SIROInspect) AND (excavat* OR "caries removal" OR "dentin removal" OR "selective caries removal") |
| **LIVIVO** | (caries OR "dentin caries" OR "carious dentin") AND (fluorescence OR "fluorescence guided" OR "fluorescence aided" OR "fluorescence aided caries excavation" OR FACE OR FIT OR SIROInspect) AND (excavat* OR "caries removal" OR "dentin removal" OR "selective caries removal") |
| **BIOSIS Citation Index** | TS=(caries OR "dentin caries" OR "carious dentin") AND TS=(fluorescence OR "fluorescence guided" OR "fluorescence aided" OR "fluorescence aided caries excavation" OR FACE OR FIT OR SIROInspect) AND TS=(excavat* OR "caries removal" OR "dentin removal" OR "selective caries removal") |

Search strategies were adapted to the syntax requirements of each database. No publication-date restriction was incorporated into the electronic search syntax. The predefined eligibility criterion excluding publications before January 1, 2000 was applied during title and abstract screening.

**Supplementary Table S3A:** Intraoperative fluorescence-guided excavation systems

| **Device / System** | **Manu-facturer** | **Head-quarters** | **Technology** | **Application Type** | **Approx. Year** |
| --- | --- | --- | --- | --- | --- |
| **FACE prototype (original system)** | University of Zurich (Buchalla, Lennon) | Zurich, Switzerland | 370–420 nm excitation + 530 nm high-pass filter | Integrated: fiber-optic light in handpiece + bur | ~2002 |
| **SIROInspect / SIROLaser Blue (FACE mode)** | Dentsply Sirona | Bensheim, Germany | ~405 nm fluorescence excitation | Combined laser + fluorescence guidance | ~2012–2015 |
| **D-Light Pro**  **(FACE mode)** | GC Corporation | Tokyo, Japan | Near-UV / ~405nm LED fluorescence | Light device + manual excavation | ~2015 |
| **SoproLife®** | Acteon Group | Mérignac, France | Blue-light fluorescence (~450 nm) camera | Visualization + conventional excavation | ~2010 |
| **VistaCam iX / VistaProof** | Dürr Dental | Bietigheim-Bissingen, Germany | ~405 nm fluorescence imaging | Visualization + separate bur | ~2009 |

**Supplementary Table S3B:** Non-FACE fluorescence-based diagnostic devices

| **DIAGNOdent**  **(Pen / Classic)** | KaVo Dental | Biberach, Germany | 655 nm laser fluorescence | Caries detection/diagnostic assessment (not intended for excavation guidance) | ~1998 |
| --- | --- | --- | --- | --- | --- |

**Note:** FACE systems provide fluorescence-based intraoperative guidance during caries excavation, whereas non-FACE diagnostic devices such as DIAGNOdent are intended primarily for caries detection and operate according to different optical principles. Their inclusion in Table S3B serves only to contextualize fluorescence-based diagnostic approaches relevant to the reviewed literature.
